# Moderation of the transgenerational transference of antenatal stress-induced anxiety

**DOI:** 10.1038/s41398-021-01383-x

**Published:** 2021-05-04

**Authors:** Or Burstein, Noam Simon, Yaarit Simchon-Tenenbaum, Moshe Rehavi, Motty Franko, Alon Shamir, Ravid Doron

**Affiliations:** 1 Department of Psychology, Bar Ilan University, Ramat Gan, Israel; 2 School of Behavioral Science, The Academic College of Tel Aviv-Yaffo, Tel Aviv-Yaffo, Israel; 3 Department of Physiology and Pharmacology, Sackler Faculty of Medicine, Tel-Aviv University, Tel-Aviv, Israel; 4 Dr. Miriam and Sheldon G. Adelson Center for the Biology of Addictive Diseases, Tel-Aviv University, Tel-Aviv, Israel; 5 Department of Psychology, Ben-Gurion University of the Negev, Be’er-Sheva, Israel; 6 Department of Education and Psychology, The Open University of Israel, Raanana, Israel; 7 Faculty of Medicine, Technion – Israel Institute of Technology, Haifa, Israel; 8 Mazor Mental Health Center, Akko, Israel

**Keywords:** Psychology, Pharmacology

## Abstract

Maternal stress has debilitating implications for both mother and child, including increased risk for anxiety. The current COVID-19 pandemic escalates these phenomena, thus, urging the need to further explore and validate feasible therapeutic options. Unlike the protracted nature of clinical studies, animal models could offer swift evidence. Prominent candidates for treatment are selective serotonin reuptake inhibitors (SSRIs) to the mother, that putatively accommodate maternal functioning, and, thereby, also protect the child. However, SSRIs might have deleterious effects. It is important to assess whether SSRIs and other pharmacotherapies can moderate the transference of anxiety by soothing maternal anxiety and to examine the extent of offspring’s exposure to the drugs via lactation. To our knowledge, the possibility that antenatal stress exacerbates lactation-driven exposure to SSRIs has not been tested yet. Thirty ICR-outbred female mice were exposed to stress during gestation and subsequently administered with either the SSRI, escitalopram, or the novel herbal candidate, shan-zha, during lactation. Upon weaning, both dams’ and pups’ anxiety-like behavior and serum escitalopram levels were assessed. The major findings of the current study show that both agents moderated the antenatal stress-induced transgenerational transference of anxiety by ameliorating dams’ anxiety. Interestingly though, pups’ exposure to escitalopram via lactation was exacerbated by antenatal stress. The latter finding provides a significant insight into the mechanism of lactation-driven exposure to xenobiotics and calls for a further consideration vis-à-vis the administration of other drugs during breastfeeding.

## Introduction

Maternal anxiety and stress have detrimental implications for both mother and child. The peripartum period is a vulnerable period for maternal anxiety disorders^1,2^, with a recent meta-analysis reporting on the disquieting global prevalence estimate of 18%^3^. Unfortunately, the current global COVID-19 pandemic escalates these phenomena—as anxiety and stress soar in the general population^4^, and specifically during peripartum^5^. Taken together, these urgently call for evidence that will promote effective and safe therapeutic strategies.

It is first important to consider the mechanism by which maternal stress impinges offspring’s development. Previous studies indicated that stress during gestation is a focal risk factor for postpartum maternal anxiety^6,7^. Further, antenatal stress was associated with increased risk for physical and mental impairments to the child^8–10^, including increased susceptibility to anxiety disorders^11–13^. Preclinical studies demonstrated a triggering of an anxiety-like phenotype in adult offspring of dams that were subjected to stress during gestation^14–17^. It remains to be elucidated whether antenatal stress triggers the transgenerational transference of anxiety from dams to pups by altering maternal anxiety in ways that affect the offspring and what could ameliorate the trans-pathogenic course during this critical time.

Several accounts suggested that through the paternal line, epigenetic means of transference could involve alterations in DNA methylation^18^ and deficiencies in microRNAs expression^19^. Experiments in rodents demonstrated that maternal care had a lasting impact on the expression of corticosteroids-related genes in the offspring, that were also associated with anxiety-like behaviors^20,21^. Similar patterns were observed in humans, suggesting that antenatal stress has a persistent and debilitating effect on children’s reactivity of the hypothalamic–pituitary–adrenal (HPA) axis, and mental health outcomes^22–24^. More specifically, both maternal ante- and postnatal anxiety strongly predicted child anxiety^23^. These accentuate the focal role of maternal anxiety in the pathogenesis of pediatric anxiety disorders. It has been suggested that maternal transference of anxiety is mediated through the effect of antenatal stress on maternal functioning^25,26^. However, the previous studies did not include a statistical method for assessing this mediation hypothesis. One of the major aims of the current treatise is to address this gap in the literature.

The prominent therapeutic candidates for postpartum anxiety are selective serotonin reuptake inhibitors (SSRIs). Their utilization has increased rapidly in recent decades^27,28^, including a prescription spike during the current pandemic^29^. Escitalopram, the *S*-enantiomer of the SSRI citalopram, is one of the most commonly prescribed drugs for anxiety and depression^30,31^, and is used by women during pregnancy and postpartum while breastfeeding^32,33^. Overall, the administration of escitalopram and other SSRIs could accommodate maternal functioning, thereby, promoting child development. However, SSRIs (including escitalopram) are excreted to some degree into the breastmilk^34^, which results in an inevitable (albeit, non-robust) infant exposure^35–37^. Serotonin plays a cardinal neurodevelopmental role; it promotes neural proliferation, migration, and differentiation, and decreases apoptosis^38^. Fluctuations in serotonergic activity during primal developmental stages might lead to long-term neural deficits and psychopathological manifestations such as anxiety, which were also observed as a sequela of fetal and neonatal SSRI exposure^39–41^. There is an ongoing debate vis-à-vis the utilization of SSRIs during breastfeeding^42,43^. This debate prompts the importance of understanding the biological grounds enabling their entry to the milk.

Myriad maternal-, infant-, and drug-related factors determine the extent to which xenobiotic agents enter the milk^44,45^. One port of entry is contingent on changes in the permeability of the tight junctions of the mammary glands, which fasten the epithelial cells together^46^. This issue is further accentuated by the critical phases for the fastening of the epithelium during mid-to-late gestation, which could be compromised by stress^47^ and maternal anxiety^48,49^. Such a compromise might widen the passage for xenobiotics into the milk^49^. To our knowledge, the possibility that antenatal stress exacerbates offspring’s SSRI exposure via lactation has not been tested yet.

Further, the ambiguity regarding peripartum SSRIs utilization is accompanied by increased interest in alternative and herbal medications^50–52^ that could broaden the therapeutic options. In recent years our group has studied a novel herbal treatment (NHT) comprised of four equal constituents (i.e., *Crataegus pinnatifida*, *Triticum aestivu*, *Lilium brownie*, and *Fructus zizyphi jujubae*). We demonstrated that NHT had anxiolytic-^53^ and antidepressant-like^54,55^ effects, comparable to these of the highly utilized SSRI escitalopram^31,32^. Exploratory studies conducted in our lab suggested that the therapeutic effects of NHT may be attributed solely to *Crataegus pinnatifida* Bge. (known in Chinese as shan-zha). Hence, examining the effects of shan-zha on maternal anxiety and offspring’s developmental outcomes could offer a novel solution for peripartum psychopathologies.

In the present study, we explored the effect of antenatal stress on anxiety-like behavior in mice upon weaning. We examined whether dams’ treatment with escitalopram or herbal treatment with shan-zha will prevent the transgenerational transference of anxiety and whether these effects are obtained through modifications of dams’ anxiety. A further aim of the study was to assess whether antenatal stress exacerbates lactation-driven exposure to escitalopram.

## Materials and Methods

### Animals

Female (*n* = 30) and male (*n* = 5) ICR-outbred mice (100 days old; Envigo RMS, Jerusalem, Israel) were housed at 22 ± 1 °C under 12 h light/dark cycles in the vivarium of the Open University. Mice had ad libitum access to rodent chow and water and were housed in standard cages with a bed of woodchips and a piece of cotton wool for enrichment^56^. All experiments were performed during the dark phase of the cycle. Male mice were used to father pups and were housed apart (five per cage) from females until mating. Female mice (three per cage) had a week of acclimation and coordination of the estrus cycle in the home cage and then were assigned for a mating cage (two females and one male per cage). Once a vaginal plug was identified, the females were transferred to a separate cage until parturition. Overall, 332 pups were delivered of which 221 (male: *n* = 123; female: *n* = 98) were randomly designated for this study. Unassessed pups were maintained with littermates to avoid additional stress and ensure similar conditions. Equal proportions of mice were utilized per litter to avoid litter effects. Sample sizes were opted based on a previous study of transgenerational effects in rodents^57^. All experiments were approved by the Academic College of Tel Aviv-Yaffo Committee for Animal Care and Use. Experiments were carried out in accordance with the NIH guidelines^58^, and efforts were made to minimize animal suffering.

### Stressor

On gestational day (GD) 15, dams were randomly assigned to either the stress (*n* = 15) or control (*n* = 15) group. Dams in the stress group were individually restrained daily in transparent plastic cylinders (30 mm diameter) under bright light (650 lux) three times a day, for 45 min until parturition^59,60^. Control dams were kept undisturbed in their home cages during gestation.

### Pharmacological agents

On postnatal day (PND) 1, dams were randomly assigned to one of three treatment groups: escitalopram (15 mg/kg), shan-zha (15 mg/kg), or control (saline). Drugs were administered daily via dorsum subcutaneous injection (to mitigate possible damage to the mammary glands that could be caused by intraperitoneal injection) until PND 21 when the period of rapid brain growth in mice is due to cease^61^. Escitalopram was kindly donated by Teva Ltd. (Petah-Tikva, Israel). Shan-zha was purchased from KPC Products (Irvine, CA, USA) as freeze-dried granules of the fruit. Drugs were dissolved in 1% dimethyl sulfoxide (DMSO) saline. This is the first assessment on the effects of chronic treatments with escitalopram and shan-zha to lactating mice dams. Therefore, doses were opted based on our previous studies with male ICR mice^55,62^, a previous report on escitalopram treatment to rat dams during gestation, and until PND 2^63^ and a pilot study, we conducted at our lab.

### Elevated plus maze (EPM)

The EPM consists of a plus maze with two black plastic closed arms and two opposite black open arms (64 cm long and 5 cm wide). The apparatus was situated 40 cm above the ground. Mice were individually placed in the center of the maze; their behavior was video recorded for 5 min and later automatically coded using the Viewer software (Biobserve GmbH, Bonn, Germany). The maze was thoroughly cleaned between sessions. Anxiety-like behavior was expressed as the percentage of time the mouse spent in the open and unprotected arms of the maze (i.e., open time/[open + enclosed time]), as previously described^64^.

### [^3^H]citalopram binding assay

Following behavioral assessments on PND 21 dams and pups’ blood samples were obtained from the facial vein into EDTA-coated tubes.

Assays were conducted by an experimenter who was blind to the animals’ group. Blood samples were centrifuged (6000×*g*; 4 °C) for 10 min and then the serum was separated and collected. To examine the presence of escitalopram in the serum, a high affinity [^3^H]citalopram binding assay was used^55^. A different set of naïve mice were decapitated and their brains were dissected on ice. The frontal cortices of the mice were disrupted with Brinkman polytron in 50 vol of ice-cold buffer (50 mM Tris-HCl, 120 mM NaCl, and 5 mM KCl; pH 7.4) and centrifuged (30,000×*g*) for 10 min (× 3). The pellet was resuspended in the same buffer to yield a final concentration of 30 mg/ml (wet weight). [^3^H]citalopram binding was determined by a standard binding assay that contained 50 μl of brain homogenate, 50 μl [^3^H]citalopram (0.5 nM; PerkinElmer Life Sciences, Boston, MA, USA), and 150 μl buffer. Serum escitalopram effect on [^3^H]citalopram binding was determined by replacing 25 μl of the buffer with 25 μl of the examined serum. After a 60 min incubation period at room temperature, the samples were washed with 3 ml ice-cold buffer (× 3) and filtered with vacuum through Whatman Glass microfiber filters GF/C (GE Healthcare Life Sciences, Chicago, IL, USA). The radioactivity was measured in a Tri-Carb 2100TR liquid scintillation counter (Packard). Specific binding was defined as the difference between total [^3^H]citalopram binding and the non-specific binding in the presence of 10 μM fluvoxamine (Sigma-Aldrich Ltd., Israel).

Essentially, the [^3^H]citalopram binding assay examined the percentage of the serotonin transporter (SERT) that was free to bind the radioactive ligand in the homogenate, as measured by *β*-counter. This percentage is an inverse measure of the serum escitalopram concentration since escitalopram competes with [^3^H]citalopram on its binding sites. Thus, a lower concentration of SERT free to bind the radioactive ligand would indicate a higher concentration of escitalopram.

### Study design

**Fig. 1:**
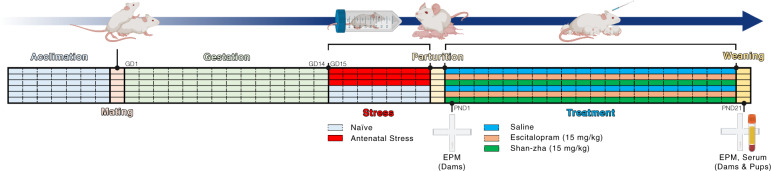
Overview of the experimental design. Each column represents one experimental day. Created with BioRender.com. EPM elevated plus maze, GD gestational day, PND postnatal day, Serum biochemical assessments of escitalopram levels in the serum.

Pregnant ICR-outbred dams were subjected to restrain stress (45 min; three times a day) from GD 15 until parturition. Following parturition, dams were examined for anxiety-like behavior (i.e., the percentage of time spent in the open arms of the maze in the EPM). Subsequently, lactating dams were treated with escitalopram (15 mg/kg), shan-zha (15 mg/kg), or saline for 3 weeks. On PND 21, dams and pups were evaluated for anxiety-like behavior. Finally, biochemical assessments weighed escitalopram concentrations in the serum in dams and pups (see Fig. 1).

### Statistical analysis

Results are expressed as mean ± SEM. Data were analyzed using independent samples *t*-tests and two-way ANOVAs as indicated, after verification that the assumption of the equality of variances between groups was met. The ANOVA was followed by Sidak post hoc analyses, except for the SERT binding that was followed by a Dunnett post hoc analysis (with escitalopram as the control). Significance was assumed as *p* < 0.05.

**Fig. 2:**
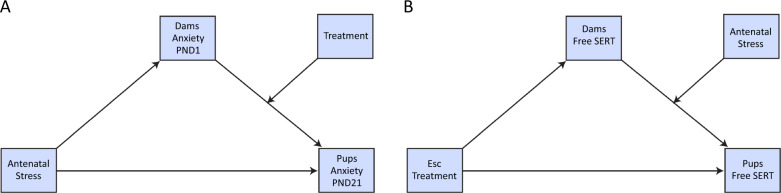
Conceptual depiction of the moderated mediation models. **A** A diagram of the hypothesized moderated mediation model on the effect of antenatal stress on pups’ anxiety-like behavior, with dams’ anxiety-like behavior on PND 1 as the mediator, and escitalopram and shan-zha treatments as moderators. **B** A diagram of the hypothesized moderated mediation model on the effect of escitalopram treatment to lactating dams on pups’ escitalopram exposure (expressed as pups’ free SERT), with dams’ escitalopram levels as the mediator, and antenatal stress as the moderator.

Conditional process analyses were conducted using the PROCESS macro^65^ for SPSS 23 (IBM, USA). This macro enables to probe hypotheses of moderated mediation, in which the effect of antecedents on outcome variables through mediators is conceived to be contingent on the presence of a moderator^65^. Indices for moderated mediation were generated, using bias-corrected 95% bootstrap confidence intervals (CIs) with 10,000 resamples. The conditional indirect effect is considered significant if the bootstrap CI does not straddle zero. PROCESS models 7 and 14 were utilized to assess whether the mediation of the effect of antenatal stress to dams on pups’ anxiety-like behavior by dams’ anxiety-like behavior on either PND 1 or PND 21 is moderated by dams’ treatment with either escitalopram or shan-zha^66^. Model 14 was also utilized to assess whether the mediation of the effect of dams’ treatment with escitalopram on pups’ serum escitalopram levels by dams’ serum escitalopram levels, is moderated by antenatal stress (i.e., that antenatal stress exacerbates the transmission of escitalopram from dams to pups via lactation). See Fig. 2 for a graphical depiction of the hypothesized moderated mediation models.

## Results

### Dams

Preliminary analyses were conducted to ascertain that the stress procedure did not cause major physical impairments to dams. The analyses assessed the effects of antenatal stress on litter size, locomotor activity, and weight; and are shown in Fig. S1 in the Supplement. These analyses indicated that the stress procedure did not cause physical damage, and, therefore, such an adverse effect is less likely to have served as an alternative explanation to the findings.

**Fig. 3:**
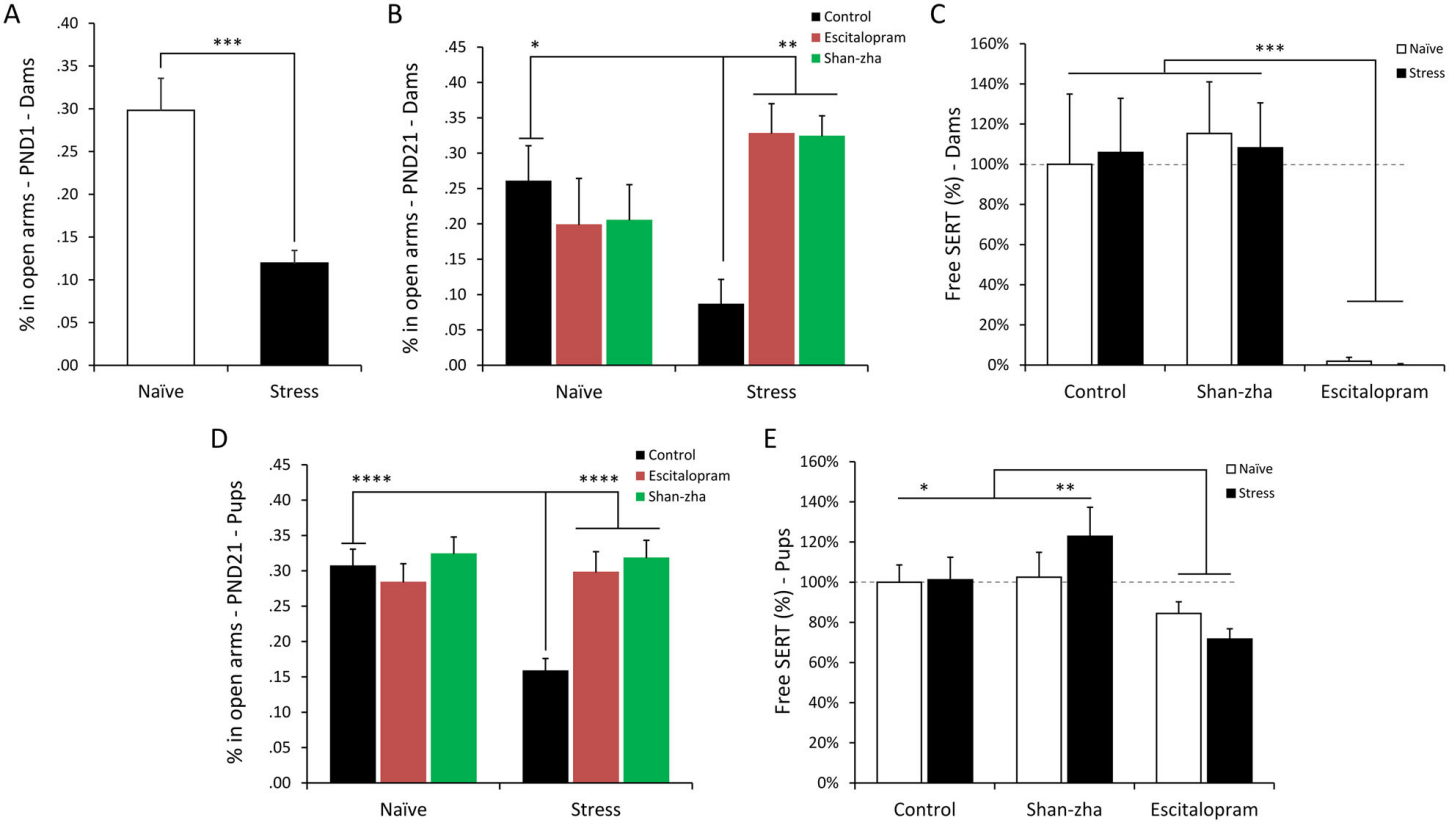
Anxiety-like behavior and escitalopram concetrations in dams and pups. **A** Antenatal stress-induced anxiety-like behaviors in dams, on PND 1. *n* = 15 dams per group. **B** Antenatal stress had a lasting effect on dams’ anxiety-like behavior, which was ameliorated by chronic treatment with either escitalopram or shan-zha. *n* = 5 dams per group. **C** Dams treated with escitalopram demonstrated reduced [^3^H]citalopram binding compared to control- and shan-zha-treated dams, indicating increased serum escitalopram level. *n* = 4 dams per group. **D** Pups that were exposed to stress during gestation demonstrated increased anxiety-like behavior on PND 21; maternal treatment with either escitalopram or shan-zha ameliorated the anxiety-like phenotype. *n* = 35–39 pups per group. **E** Pups of dams that were treated with escitalopram demonstrated increased serum escitalopram levels. *n* = 14–16 pups per group. Dashed lines represent means of reference groups (i.e., control-naïve groups). *p < 0.05, **p < 0.01, ***p < 0.001, ****p < 0.0001.

Exposure to antenatal stress-induced an anxiety-like phenotype in dams on PND 1 (*t*_(28)_ = 4.44, *p* = 0.0001; Fig. 3A). Analysis of anxiety-like behavior on PND 21 revealed a significant stress × treatment interaction effect (*F*_(2,24)_ = 6.96, *p* = 0.004; Fig. 3B). Post hoc analysis revealed that stress had a durable effect on dams’ anxiety-like behavior (*p* = 0.014). However, chronic treatments with either escitalopram or shan-zha ameliorated the antenatal stress-induced deficits (*p* < 0.01 in both contrasts).

Analysis of dams’ free SERT revealed a significant effect for treatment (*F*_(2,18)_ = 14.61, *p* = 0.0002; Fig. 3C), suggesting increased serum levels of escitalopram following its chronic administration (*p* < 0.001).

### Pups

No differences between male and female pups were observed in the utilized measures; thus, both groups were considered together for further analyses (for further details see Table S1 and Table S2 in the Supplement).

Analysis of pups’ anxiety-like behavior on PND 21 revealed a significant stress × treatment interaction effect (*F*_(2,215)_ = 6.91, *p* = 0.001; Fig. 3D). Post hoc analysis revealed that pups that were exposed to stress during gestation and nursed by antenatally-stressed dams spent less time in the open arms of the maze compared to controls (*p* < 0.0001). Interestingly though, pups that were nursed by either escitalopram- or shan-zha-treated antenatally-stressed dams demonstrated equivalent behavior to controls.

Analysis of pups’ free SERT revealed a significant effect for treatment (*F*_(2,84)_ = 5.6, *p* = 0.005; Fig. 3E), suggesting increased serum levels of escitalopram in pups that were nursed by escitalopram-treated dams (*p* < 0.05). This indicates that escitalopram was excreted into dams’ milk and transferred to pups via lactation. An additional Sidak post hoc analysis did not find a significant difference between the control and shan-zha groups (*p* = 0.58), suggesting that shan-zha treatment to dams had no impact on pups’ serum escitalopram levels.

### Transgenerational transference of anxiety

**Fig. 4:**
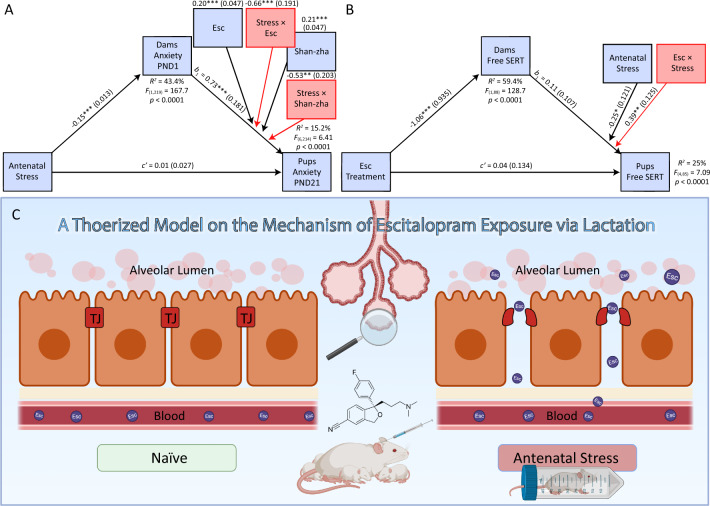
Moderated mediation models. **A** Antenatal stress led to a significant decrease in the time dams spent in the open arms of the maze on PND 1 (*a*_1_ = −0.15, *p* < 0.0001); chronic treatment with either escitalopram (*b*_4_ = −0.66, *p* = 0.0007) or shan-zha (*b*_5_ = −0.53, *p* = 0.009) to lactating dams that were exposed to antenatal stress significantly moderated the association between dams’ and pups’ anxiety. Higher values on the anxiety indices indicate diminished anxiety-like-behavior. *N* = 221. **B** Chronic treatment with escitalopram led to a significant decrease in dams’ free SERT (*a*_1_ = −1.06, *p* < 0.0001); exposure to antenatal stress served as a precursor for the association between dams’ and pups’ free SERT (*b*_4_ = 0.39, *p* = 0.002), suggesting that antenatal stress exacerbated the transfer of escitalopram from dams to pups via lactation. *N* = 90. **C** A graphical depiction of the hypothesized mechanism by which antenatal stress might exacerbate pups’ exposure to escitalopram; putatively, via durable alterations of the permeability of the mammary glandular epithelium that is instigated in the critical stage of mid-to-late gestation. Created with BioRender.com. Esc escitalopram, TJ tight junction. *p < 0.05, **p < 0.01, ***p < 0.001.

A conditional process analysis indicated that the effect of antenatal stress on pups’ anxiety-like behavior was mediated by dams’ anxiety-like behavior on PND 1. Further, both escitalopram and shan-zha moderated the stress-induced transference of anxiety from dams to pups by soothing dams’ anxiety from PND 1 (escitalopram: *B* = −0.66, *p* = 0.0007; shan-zha: *B* = −0.53, *p* = 0.009). The effect of antenatal stress on pups’ anxiety was solely related to modifications of dams’ anxiety, with no significant direct effect (*c*′ = 0.01, *p* = 0.64). The indices of moderated mediation suggested that dams’ treatment with either escitalopram (95% Boot CI, 0.051 to 0.186) or shan-zha (95% Boot CI, 0.024 to 0.167) ameliorated the stress-induced transference of anxiety to pups when compared to controls (Fig. 4A; for further details see Table S3). An additional model involving dams’ anxiety-like behavior on PND 21 as the mediator showed equivalent results (this analysis is delineated in Table S4 and Fig. S2 in the Supplement); thus, reinforcing the validity of the moderated mediation premise.

### Antenatal stress exacerbates pups’ exposure to escitalopram via lactation

A conditional process analysis indicated that the effect of treatment with escitalopram to dams on pups’ escitalopram exposure was fully mediated by escitalopram levels in dams’ serum, with no significant direct effect (*c*′ = 0.04, *p* = 0.78). However, the exposure via lactation was only significant in pups that were fed by antenatally-stressed dams (95% Boot CI, −0.78 to −0.28) and not by naïve dams (95% Boot CI, −0.37 to 0.10). The index of moderated mediation (Index = −0.417; 95% Boot CI, −0.64 to −0.17) suggested that dams’ exposure to antenatal stress catalyzed the lactation-driven transmission of escitalopram to pups (Fig. 4B; for further details see Table S5), putatively by altering the permeability of the mammary gland.

## Discussion

The current study brings about two noteworthy findings. First, it demonstrates that the mechanism responsible for annulling the transgenerational transference of anxiety is related to the ability of both escitalopram and shan-zha to modify and soothe dams’ anxiety-like behavior. Second, the findings demonstrate that antenatal stress exacerbates pups’ exposure to escitalopram.

Previous studies demonstrated increments in anxiety-like behaviors in offspring that were exposed to antenatal stress^15,67–69^. These studies reported on behavioral abnormalities in adulthood, while our findings imply that abnormalities are already evident upon weaning. A recent meta-analysis reported that ~6.5% of children and adolescents worldwide suffer from any anxiety disorder^70^; the risk is much higher when the child is nurtured by a mother who struggles with an anxiety disorder^11,23^. The current findings suggest that maternal anxiety plays a major role in child development and that addressing it with appropriate treatment could redirect the child’s developmental course towards a more salubrious path, and accentuate the significance of early clinical evaluation and intervention for susceptible individuals.

Escitalopram treatment to dams prevented the antenatal stress-induced anxiety-like phenotype in both dams and offspring. Limited research was conducted thus far to assess the effects of SSRIs administration to lactating dams on offspring outcomes. Zohar and colleagues^57^ showed that citalopram treatment from GD 7 to PND 21 normalized rat dams’, but did not avert pups’ antenatal stress-induced anxiety-like behaviors. A major limitation of this study is that the initiation of the SSRI treatment was during gestation, suggesting a much more severe impingement to serotonergic signaling, due to the magnitude of fetus exposure (when compared to lactation-based exposure)^35^. In the current study, escitalopram was administered to dams only after parturition. This possibly caused lesser disruption to brain development, while simultaneously improved dams’ and subsequently pups’ outcomes.

To our knowledge, this is the first portrayal of the mechanism by which antenatal stress might exacerbate the transfer of escitalopram to pups. Based on this finding, we formulated a model that could broaden the understanding of lactation-driven exposure to xenobiotics. According to the model, exposure to stress during critical periods of gestation might impair the formation of the mammary gland permeability, thus, potentiating offspring’s exposure to SSRIs via lactation (Fig. 4C). During mid-late-gestational phases (i.e., GD 14 to 20) major transformations take place in the mice mammary gland, including epithelium cell proliferation, alveolar differentiation, and decrease in adipose tissue. These changes are comparable to the ones observed in humans^71^. During gestation the mammary tight junctions go through a biomolecular process that facilitates their closure, reaching an almost impermeable state near parturition^46^. Glucocorticoids are focal modulators of the closure of the tight junctions; putatively, increased secretion of glucocorticoids near parturition enables their closure^72,73^. We speculate that disruption of the glucocorticoid equilibrium (e.g., via exposure to stress) might impair the typical formation of the mammary tight junctions expected in late pregnancy. Nonetheless, in the current study, no direct assessment of the mammary gland permeability was performed, and, therefore, this model should be regarded as a hypothesized model; it is possible that other explanations, such as antenatal stress-induced alterations in pups’ drug metabolism^74^, played a further role in the excessive levels of escitalopram. Taken together, the presented model calls for validation in future research, that will also weigh the effects of stress on glucocorticoid levels during the sensitive period of mid-to-late gestation. This model might have important implications when weighing pharmacotherapies for susceptible breastfeeding mothers (e.g., mothers that were exposed to stress during pregnancy or suffer from anxiety), that are highly relevant due to the COVID-19-related intensification of maternal anxiety; and calls for evaluations of the interplay between stress and other drugs that are currently being utilized during breastfeeding.

The moderated mediation premise of the current study is based on the emerging utilization of such statistical procedures in various disciplines to assess how and when effects are crystalized^65,75,76^. However, it has been previously shown that some studies make inferences of mediation and moderation without performing the suitable statistical tests^77^. We suggest that the utilization of conditional process analyses for elucidating pathophysiological mechanisms could be highly constructive for preclinical research in biological psychiatry. Important insights were gained by its usage in the current study, including the transgenerational anxiolytic effect found for shan-zha.

Shan-zha treatment to dams paralleled the anxiolytic effect of escitalopram. This finding is consistent with the growing body of research in our lab^53,54^. Shan-zha has been applied for many centuries in traditional Chinese medicine for treating fluctuations in mood, body temperature, and energy^78^. Previous studies demonstrated both in vivo and in vitro, that shan-zha elicits anti-inflammatory^79^ and antioxidant^79^ effects; these are putatively related to its phenolic constituents^80,81^: hyperoside^82^, chlorogenic acid^82,83^, quercetin^82,84^, and ursolic acid^85^. Recently, Lim and colleagues^83^ suggested that these qualities of shan-zha may underlie its psychopharmacological potential; they demonstrated that shan-zha treatment prevented the stress hormone-induced retraction in hippocampal dendritic spine density and induced an antidepressant-like effect in ICR mice. As such morphological alterations in the hippocampus are highly implicated in the etiology of anxiety disorders^86^, this neuroprotective effect may explain the therapeutic mechanism of shan-zha. To our knowledge, this is the first study to assess the effect of shan-zha on anxiety-like behavior, and it brings about promising evidence involving the moderation of pups’ anxiety-like behavior, via the modification of dams’ anxiety.

A major limitation of the current study is that although we conclude that the modification in pups’ behavior was a consequence of the changes in maternal functioning, no direct assessment of maternal care was included. However, various ante- and post-natal stress procedures in mice were previously shown to also impair maternal behavior^87–91^. Further, maternal anxiety is an imperative antecedent for subsequent anxiety disorders in children^23^, and the targeting of anxiety-driven parenting behaviors was advocated as a crucial factor in promoting better treatment outcomes for children suffering from anxiety disorders^92,93^. The current design assimilated these notions, suggesting that maternal anxiety per se has a prominent role in determining anxiety-related pathologies in offspring.

Whether to utilize pharmacological treatments for maternal anxiety and depression during breastfeeding is still a genuine clinical dilemma. The application of pharmacotherapies is supported by empirical findings regarding the damaging impact of untreated postpartum psychiatric illness on infants’ psychomotor, cognitive, social, and emotional development^94–97^. On the other hand, maternal SSRI treatment during breastfeeding was associated with adverse neurodevelopmental outcomes^98,99^. It is debated whether the effects of maternal pharmacological interventions on child development are obtained through modifications of maternal care or by exposure to the drug while breastfeeding. We confirmed that escitalopram was significantly evident in the serum of pups of antenatally-stressed dams; however, we found that the amelioration of the transgenerational transference of anxiety was obtained by the soothing of maternal anxiety (and not by lactation-driven drug exposure). Thus, the current study generally supports psychopharmacological targeting of maternal anxiety, while acknowledges that maternal stress and anxiety might facilitate infant exposure to SSRIs.

In conclusion, our results provide support to the notion that exposure to antenatal stress increases anxiety-likes behaviors in offspring, through intensification of maternal anxiety. Dams’ treatment with either escitalopram or shan-zha was effective in preventing the stress-induced transference of anxiety from dams to pups. Even though it was established that antenatal stress potentiated the transfer of escitalopram to pups, there were no indications of harm. Further studies should extend our understanding of these questions, to enable more informed reasoning when weighing pharmacological interventions for postpartum anxiety, while bearing in mind both mother and child.

## Supplementary information

Supplement

## References

[1] Wenzel A, Haugen EN, Jackson LC, Brendle JR (2005). Anxiety symptoms and disorders at eight weeks postpartum. J. Anxiety Disord..

[2] Brockington I (2004). Diagnosis and management of post-partum disorders: a review. World Psychiatry.

[3] Fawcett, E. J., Fairbrother, N., Cox, M. L., White, I. R. & Fawcett, J. M. The prevalence of anxiety disorders during pregnancy and the postpartum period: a multivariate Bayesian meta-analysis. *J. Clin. Psychiatry*10.4088/JCP.18r12527 (2019). 10.4088/JCP.18r12527 PMC6839961 31347796

[4] Salari, N. et al. Prevalence of stress, anxiety, depression among the general population during the COVID-19 pandemic: a systematic review and meta-analysis. *Global. Health*10.1186/s12992-020-00589-w (2020). 10.1186/s12992-020-00589-w PMC7338126 32631403

[5] Davenport, M. H. et al. Moms are not ok: COVID-19 and maternal mental health. *Front. Glob. Women’s Health*10.3389/fgwh.2020.00001 (2020). 10.3389/fgwh.2020.00001 PMC8593957 34816146

[6] Dennis, C. L., Falah-Hassani, K., Brown, H. K. & Vigod, S. N. Identifying women at risk for postpartum anxiety: a prospective population-based study. *Acta Psychiatr. Scand*. 10.1111/acps.12648 (2016). 10.1111/acps.12648 27639034

[7] Rice, F. et al. The links between prenatal stress and offspring development and psychopathology: disentangling environmental and inherited influences. *Psychol. Med.*10.1017/S0033291709005911 (2010). 10.1017/S0033291709005911 PMC2830085 19476689

[8] Wadhwa PD (2005). Psychoneuroendocrine processes in human pregnancy influence fetal development and health. Psychoneuroendocrinology.

[9] Seckl JR, Holmes MC (2007). Mechanisms of disease: glucocorticoids, their placental metabolism and fetal ‘programming’ of adult pathophysiology. Nat. Clin. Pr. Endocrinol. Metab..

[10] Talge NM, Neal C, Glover V (2007). Antenatal maternal stress and long-term effects on child neurodevelopment: how and why?. J. Child Psychol. Psychiatry Allied Discip..

[11] Van Den Bergh BRH, Marcoen A (2004). High antenatal maternal anxiety is related to ADHD symptoms, externalizing problems, and anxiety in 8- and 9-year-olds. Child Dev..

[12] Davis EP, Sandman CA (2012). Prenatal psychobiological predictors of anxiety risk in preadolescent children. Psychoneuroendocrinology.

[13] Glover V (2014). Maternal depression, anxiety and stress during pregnancy and child outcome; What needs to be done. Best. Pr. Res. Clin. Obstet. Gynaecol..

[14] Kapoor A, Matthews SG (2005). Short periods of prenatal stress affect growth, behaviour and hypothalamo-pituitary-adrenal axis activity in male guinea pig offspring. J. Physiol..

[15] Darnaudéry M, Maccari S (2008). Epigenetic programming of the stress response in male and female rats by prenatal restraint stress. Brain Res. Rev..

[16] Weinstock M (2008). The long-term behavioural consequences of prenatal stress. Neurosci. Biobehav. Rev..

[17] Marco EM, MacRì S, Laviola G (2011). Critical age windows for neurodevelopmental psychiatric disorders: evidence from animal models. Neurotox. Res..

[18] Franklin, T. B. et al. Epigenetic transmission of the impact of early stress across generations. *Biol. Psychiatry*10.1016/j.biopsych.2010.05.036 (2010). 10.1016/j.biopsych.2010.05.036 20673872

[19] Gapp, K. et al. Implication of sperm RNAs in transgenerational inheritance of the effects of early trauma in mice. *Nat. Neurosci*. 10.1038/nn.3695 (2014). 10.1038/nn.3695 PMC4333222 24728267

[20] Liu, D. et al. Maternal care, hippocampal glucocorticoid receptors, and hypothalamic- pituitary-adrenal responses to stress. *Science*10.1126/science.277.5332.1659 (1997). 10.1126/science.277.5332.1659 9287218

[21] Weaver, I. C. G., Meaney, M. J. & Szyf, M. Maternal care effects on the hippocampal transcriptome and anxiety-mediated behaviors in the offspring that are reversible in adulthood. *Proc. Natl Acad. Sci. USA*10.1073/pnas.0507526103 (2006). 10.1073/pnas.0507526103 PMC1413873 16484373

[22] Essex, M. J., Klein, M. H., Cho, E. & Kalin, N. H. Maternal stress beginning in infancy may sensitize children to later stress exposure: effects on cortisol and behavior. *Biol. Psychiatry*10.1016/S0006-3223(02)01553-6 (2002). 10.1016/s0006-3223(02)01553-6 12372649

[23] Sharp, H., Hill, J., Hellie,r J. & Pickles, A. Maternal antenatal anxiety, postnatal stroking and emotional problems in children: outcomes predicted from pre- and postnatal programming hypotheses. *Psychol. Med*. 10.1017/S0033291714001342 (2015). 10.1017/S0033291714001342 PMC4301199 25068652

[24] Madigan, S. et al. A meta-analysis of maternal prenatal depression and anxiety on child socioemotional development. *J. Am. Acad. Child Adolesc. Psychiatry*. 10.1016/j.jaac.2018.06.012 (2018). 10.1016/j.jaac.2018.06.012 30196868

[25] Walker, A. K., Hawkins, G., Sominsky, L. & Hodgson, D. M. Transgenerational transmission of anxiety induced by neonatal exposure to lipopolysaccharide: implications for male and female germ lines. *Psychoneuroendocrinology*10.1016/j.psyneuen.2012.01.005 (2012). 10.1016/j.psyneuen.2012.01.005 22342246

[26] Penteado, S. H. W. et al. Prenatal lipopolysaccharide disrupts maternal behavior, reduces nest odor preference in pups, and induces anxiety: studies of F1 and F2 generations. *Eur. J. Pharmacol*. 10.1016/j.ejphar.2014.05.058 (2014). 10.1016/j.ejphar.2014.05.058 24927995

[27] Molenaar, N. M. et al. The international prevalence of antidepressant use before, during, and after pregnancy: a systematic review and meta-analysis of timing, type of prescriptions and geographical variability. *J. Affect. Disord.*10.1016/j.jad.2019.12.014 (2020). 10.1016/j.jad.2019.12.014 31846905

[28] Sun Y (2019). Trend of antidepressants before, during, and after pregnancy across two decades—A population-based study. Brain Behav..

[29] Express Scripts. America’s State of Mind Report: U.S. trends in medication use for depression, anxiety and insomnia. https://www.express-scripts.com/corporate/americas-state-of-mind-report. (2020).

[30] Höschl C, Svestka J (2008). Escitalopram for the treatment of major depression and anxiety disorders. Expert Rev. Neurother..

[31] Cipriani A (2009). Comparative efficacy and acceptability of 12 new-generation antidepressants: a multiple-treatments meta-analysis. Lancet.

[32] Bellantuono C, Bozzi F, Orsolini L, Catena-Dell’Osso M (2012). The safety of escitalopram during pregnancy and breastfeeding: a comprehensive review. Hum. Psychopharmacol..

[33] Delaney, S. R. et al. Predicting escitalopram exposure to breastfeeding infants: integrating analytical and in silico techniques. *Clin. Pharmacokinet.*10.1007/s40262-018-0657-2 (2018). 10.1007/s40262-018-0657-2 29651785

[34] Rampono J (2009). Placental transfer of SSRI and SNRI antidepressants and effects on the neonate. Pharmacopsychiatry.

[35] Capello CF (2011). Serotonin transporter occupancy in rats exposed to serotonin reuptake inhibitors in utero or via breast milk. J. Pharm. Exp. Ther..

[36] Orsolini L, Bellantuono C (2015). Serotonin reuptake inhibitors and breastfeeding: a systematic review. Hum. Psychopharmacol..

[37] Drug and Lactation Database. *Drug and Lactation Database* (*LactMed*) (National Library of Medicine, 2020).

[38] Vitalis, T. & Parnavelas, J. G. The role of serotonin in early cortical development. *Dev. Neurosci*. 10.1159/000072272 (2003). 10.1159/000072272 12966221

[39] Ansorge, M. S., Zhou, M., Lira, A., Hen, R., & Gingrich, J. A. Early-life blockade of the 5-HT transporter alters emotional behavior in adult mice. *Science*10.1126/science.1101678 (2004). 10.1126/science.1101678 15514160

[40] Olivier, J. D. A. et al. Fluoxetine administration to pregnant rats increases anxiety-related behavior in the offspring. *Psychopharmacology*10.1007/s00213-011-2299-z (2011). 10.1007/s00213-011-2299-z 21487650

[41] Lugo-Candelas, C. et al. Associations between brain structure and connectivity in infants and exposure to selective serotonin reuptake inhibitors during pregnancy. *JAMA Pediatr*. 10.1001/jamapediatrics.2017.5227 (2018). 10.1001/jamapediatrics.2017.5227 PMC6137537 29630692

[42] Fischer Fumeaux CJ (2019). Risk-benefit balance assessment of SSRI antidepressant use during pregnancy and lactation based on best available evidence – an update. Expert Opin. Drug Saf..

[43] Chad L, Pupco A, Bozzo P, Koren G (2013). Update on antidepressant use during breastfeeding. Can. Fam. Physician.

[44] Howard CR, Lawrence RA (1999). Drugs and breastfeeding. Clin. Perinatol..

[45] McManaman, J. L. & Neville, M. C. Mammary physiology and milk secretion. *Adv. Drug Deliv. Rev*. 10.1016/S0169-409X(03)00033-4 (2003). 10.1016/s0169-409x(03)00033-4 12706546

[46] Nguyen, D. A. D. & Neville, M. C. Tight junction regulation in the mammary gland. *J. Mammary Gland Biol. Neoplasia*10.1023/A:1018707309361 (1998). 10.1023/a:1018707309361 10819511

[47] Stelwagen, K., Hopster, H., Van Der Werf, J. T. N. & Blokhuis, H. J. Short communication: effects of isolation stress on mammary tight junctions in lactating dairy cows. *J. Dairy Sci*. 10.3168/jds.S0022-0302(00)74853-3 (2000). 10.3168/jds.S0022-0302(00)74853-3 10659962

[48] Flores-Quijano, M. E. et al. Risk for postpartum depression, breastfeeding practices, and mammary gland permeability. *J. Hum. Lact*. 10.1177/0890334407310587 (2008). 10.1177/0890334407310587 18281356

[49] Ozbek, A. et al. Maternal psychosocial aspects in hypernatremic dehydration with high sodium concentrations in breast milk: a case-control study. *J. Paediatr. Child Health*10.1111/j.1440-1754.2007.01208.x (2008). 10.1111/j.1440-1754.2007.01208.x 17854413

[50] Allaire AD, Moos MK, Wells SR (2000). Complementary and alternative medicine in pregnancy: a survey of North Carolina certified nurse-midwives. Obstet. Gynecol..

[51] Pan Y-J, Cheng I-C, Yeh L-L, Cho Y-M, Feng J (2013). Utilization of traditional Chinese medicine in patients treated for depression: a population-based study in Taiwan. Complement Ther. Med..

[52] Kessler RC (2001). The use of complementary and alternative therapies to treat anxiety and depression in the United States. Am. J. Psychiatry.

[53] Doron R (2018). GABAA receptor density is not altered by a novel herbal anxiolytic treatment. J. Mol. Neurosci..

[54] Burstein, O. et al. Escitalopram and NHT normalized stress-induced anhedonia and molecular neuroadaptations in a mouse model of depression. *PLoS ONE*10.1371/journal.pone.0188043 (2017). 10.1371/journal.pone.0188043 PMC5687745 29141007

[55] Doron R (2014). A novel herbal treatment reduces depressive-like behaviors and increases BDNF levels in the brain of stressed mice. Life Sci..

[56] Chourbaji S, Brandwein C, Gass P (2011). Altering BDNF expression by genetics and/or environment: impact for emotional and depression-like behaviour in laboratory mice. Neurosci. Biobehav. Rev..

[57] Zohar I, Shoham S, Weinstock M (2016). Perinatal citalopram does not prevent the effect of prenatal stress on anxiety, depressive-like behaviour and serotonergic transmission in adult rat offspring. Eur. J. Neurosci..

[58] National Research Council. *Guide for the Care and Use of Laboratory Animals* (National Academies Press, 2011). 21595115

[59] Ward IL, Weisz J (1984). Differential effects of maternal stress on circulating levels of corticosterone, progesterone, and testosterone in male and female rat fetuses and their mothers. Endocrinology.

[60] Van Den Hove DLA (2005). Prenatal restraint stress and long-term affective consequences. Dev. Neurosci..

[61] Johansson N, Eriksson P, Viberg H (2009). Neonatal exposure to PFOS and PFOA in mice results in changes in proteins which are important for neuronal growth and synaptogenesis in the developing brain. Toxicol. Sci..

[62] Doron, R. et al. Cerebral MAO activity is not altered by a novel herbal antidepressant treatment. *J. Mol. Neurosci.*10.1007/s12031-019-01366-0 (2019). 10.1007/s12031-019-01366-0 31290092

[63] Bourke, C. H., Stowe, Z. N., Neigh, G. N., Olson, D. E. & Owens, M. J. Prenatal exposure to escitalopram and/or stress in rats produces limited effects on endocrine, behavioral, or gene expression measures in adult male rats. *Neurotoxicol. Teratol.*10.1016/j.ntt.2013.07.008 (2013). 10.1016/j.ntt.2013.07.008 PMC3795959 23906943

[64] Carobrez, A. P., Kincheski, G. C. & Bertoglio, L. J. *Encyclopedia of Psychopharmacology* (Springer, 2015).

[65] Hayes, A. F. *Introduction to Mediation, Moderation, and Conditional Process Analysis*: *A Regression-Based Approach* (Guilford Press, 2018).

[66] Hayes, A. F. An index and test of linear moderated mediation. *Multivariate Behav. Res.*10.1080/00273171.2014.962683 (2015). 10.1080/00273171.2014.962683 26609740

[67] Morley-Fletcher S (2011). Chronic agomelatine treatment corrects behavioral, cellular, and biochemical abnormalities induced by prenatal stress in rats. Psychopharmacol..

[68] Wang Y (2015). Sexual differences in long-term effects of prenatal chronic mild stress on anxiety-like behavior and stress-induced regional glutamate receptor expression in rat offspring. Int. J. Dev. Neurosci..

[69] Marrocco J (2014). The effects of antidepressant treatment in prenatally stressed rats support the glutamatergic hypothesis of stress-related disorders. J. Neurosci..

[70] Polanczyk GV, Salum GA, Sugaya LS, Caye A, Rohde LA (2015). Annual research review: a meta-analysis of the worldwide prevalence of mental disorders in children and adolescents. J. Child Psychol. Psychiatry Allied Discip..

[71] Cardiff, R. D. et al. *Comparative Anatomy and Histology* (Elsevier, 2018).

[72] Zettl, K. S. et al. Glucocorticoid-induced formation of tight junctions in mouse mammary epithelial cells in vitro. *Proc. Natl Acad. Sci. USA*10.1073/pnas.89.19.9069 (1992). 10.1073/pnas.89.19.9069 PMC50066 1409603

[73] Stelwagen, K., McFadden, H. A. & Demmer, J. Prolactin, alone or in combination with glucocorticoids, enhances tight junction formation and expression of the tight junction protein occludin in mammary cells. *Mol. Cell Endocrinol*. 10.1016/S0303-7207(99)00145-8 (1999). 10.1016/s0303-7207(99)00145-8 10612423

[74] Gurnot, C. et al. Prenatal antidepressant exposure associated with CYP2E1 DNA methylation change in neonates. *Epigenetics*10.1080/15592294.2015.1026031 (2015). 10.1080/15592294.2015.1026031 PMC4622680 25891251

[75] Rucker, D. D., Preacher, K. J., Tormala, Z. L. & Petty, R. E. Mediation analysis in social psychology: current practices and new recommendations. *Soc. Personal Psychol. Compass*10.1111/j.1751-9004.2011.00355.x (2011).

[76] Rungtusanatham, M., Miller, J. W. & Boyer, K. K. Theorizing, testing, and concluding for mediation in SCM research: tutorial and procedural recommendations. *J. Oper. Manag*. 10.1016/j.jom.2014.01.002 (2014).

[77] Memon, M. A., Cheah, J.-H., Ramayah, T., Ting, H. & Chuah, F. Mediation analysis: issues and recommendations. *J. Appl. Struct. Equ. Model*10.47263/jasem.2(1)01 (2018).

[78] Ross, J. *Combining Western Herbs and Chinese Medicine: Principles, Practice, and Materia Medica* (Greenfields Press, 2003).

[79] Li, C. et al. Comparison of Crataegus pinnatifida Bunge var. typica Schneider and C. pinnatifida Bunge fruits for antioxidant, anti-α-glucosidase, and anti-inflammatory activities. *Food Sci. Biotechnol.*10.1007/s10068-010-0108-9 (2010).

[80] Liu, P., Yang, B. & Kallio, H. Characterization of phenolic compounds in Chinese hawthorn (Crataegus pinnatifida Bge. var. major) fruit by high performance liquid chromatography-electrospray ionization mass spectrometry. *Food Chem*. 10.1016/j.foodchem.2010.02.002 (2010). 10.1016/j.foodchem.2011.01.103 25214140

[81] Wu J., Peng W., Qin R., Zhou H. Crataegus pinnatifida: chemical constituents, pharmacology, and potential applications. *Molecules*10.3390/molecules19021685 (2014). 10.3390/molecules19021685 PMC6271784 24487567

[82] Zhang, L. L., Zhang, L. F. & Xu, J. G. Chemical composition, antibacterial activity and action mechanism of different extracts from hawthorn (Crataegus pinnatifida Bge.). *Sci. Rep.*10.1038/s41598-020-65802-7 (2020). 10.1038/s41598-020-65802-7 PMC7264281 32483369

[83] Lim, D. W. et al. Chlorogenic acid from hawthorn berry (Crataegus pinnatifida fruit) prevents stress hormone-induced depressive behavior, through monoamine oxidase B-reactive oxygen species signaling in hippocampal astrocytes of mice. *Mol. Nutr. Food Res.*10.1002/mnfr.201800029 (2018). 10.1002/mnfr.201800029 29893510

[84] Merzoug, S., Toumi, M. L., Tahraoui, A. Quercetin mitigates Adriamycin-induced anxiety- and depression-like behaviors, immune dysfunction, and brain oxidative stress in rats. *Naunyn Schmiedebergs Arch. Pharmacol*. 10.1007/s00210-014-1008-y (2014). 10.1007/s00210-014-1008-y 24947870

[85] Colla, A. R. S., Rosa, J. M., Cunha, M. P. & Rodrigues, A. L. S. Anxiolytic-like effects of ursolic acid in mice. *Eur. J. Pharmacol*. 10.1016/j.ejphar.2015.03.077 (2015). 10.1016/j.ejphar.2015.03.077 25861934

[86] Leuner, B. & Shors, T. J. Stress, anxiety, and dendritic spines: what are the connections? *Neuroscience*10.1016/j.neuroscience.2012.04.021 (2013). 10.1016/j.neuroscience.2012.04.021 22522470

[87] Pardon, M. C., Gérardin, P., Joubert, C., Pérez-Diaz, F. & Cohen-Salmon, C. Influence of prepartum chronic ultramild stress on maternal pup care behavior in mice. *Biol. Psychiatry*10.1016/S0006-3223(99)00253-X (2000). 10.1016/s0006-3223(99)00253-x 10807958

[88] Meek, L. R., Dittel, P. L., Sheehan, M. C., Chan, J. Y. & Kjolhaug, S. R. Effects of stress during pregnancy on maternal behavior in mice. *Physiol. Behav*. 10.1016/S0031-9384(00)00431-5 (2001). 10.1016/s0031-9384(00)00431-5 11282130

[89] Yamada, K., Santo-Yamadal, Y. & Wada, K. Restraint stress impaired maternal behavior in female mice lacking the neuromedin B receptor (NMB-R) gene. *Neurosci. Lett*. 10.1016/S0304-3940(02)00771-1 (2002). 10.1016/s0304-3940(02)00771-1 12231437

[90] St-Cyr, S. & McGowan, P. O. Programming of stress-related behavior and epigenetic neural gene regulation in mice offspring through maternal exposure to predator odor. *Front. Behav. Neurosci.*10.3389/fnbeh.2015.00145 (2015). 10.3389/fnbeh.2015.00145 PMC4450170 26082698

[91] Nephew, B. C. et al. Intergenerational accumulation of impairments in maternal behavior following postnatal social stress. *Psychoneuroendocrinology*10.1016/j.psyneuen.2017.05.011 (2017). 10.1016/j.psyneuen.2017.05.011 PMC6390956 28528143

[92] Creswell, C., Willetts, L., Murray, L., Singhal, M. & Cooper, P. Treatment of child anxiety: an exploratory study of the role of maternal anxiety and behaviours in treatment outcome. *Clin. Psychol. Psychother*. 10.1002/cpp.559 (2008). 10.1002/cpp.559 19115426

[93] Kerns, C. E., Pincus, D. B., McLaughlin, K. A. & Comer, J. S. Maternal emotion regulation during child distress, child anxiety accommodation, and links between maternal and child anxiety. *J. Anxiety Disord*. 10.1016/j.janxdis.2017.05.002 (2017). 10.1016/j.janxdis.2017.05.002 28577415

[94] Murray L, Fiori-Cowley A, Hooper R, Cooper P (1996). The impact of postnatal depression and associated adversity on early mother-infant interactions and later infant outcome. Child Dev..

[95] Cornish AM (2005). Postnatal depression and infant cognitive and motor development in the second postnatal year: the impact of depression chronicity and infant gender. Infant Behav. Dev..

[96] Barker ED, Jaffee SR, Uher R, Maughan B (2011). The contribution of prenatal and postnatal maternal anxiety and depression to child maladjustment. Depress. Anxiety.

[97] Coplan RJ, O’Neil K, Arbeau KA (2005). Maternal anxiety during and after pregnancy and infant temperament at three months of age. J. Prenat. Perinat. Psychol. Health.

[98] Lanza di Scalea T, Wisner KL (2009). Antidepressant medication use during breastfeeding. Clin. Obs. Gynecol..

[99] Gentile S. & Fusco, M. L. Antidepressants During Breastfeeding. in *Perinatal Psychopharmacology*, Chapter 6, 99–113 (eds Uguz, F. & Orsolini,L.) (Springer International Publishing, 2019) 10.1007/978-3-319-92919-4_6.

