# Supplementary material for: Moderation of the transgenerational transference of antenatal stress-induced anxiety
Source: Transl Psychiatry. 2021 May 4;11:268. doi: 10.1038/s41398-021-01383-x (PMC8094124; doi:10.1038/s41398-021-01383-x)
Supplement: Supplementary file 1 — Supplement [file 41398_2021_1383_MOESM1_ESM.docx]

**Appendix A**

**The effects of antenatal stress on dams' physiological indices**

Analyses were carried out to ascertain that the stress manipulation did not cause major physical impairments to dams, which could have served as a possible alternative explanation to the results.

*Litter size*

An independent samples *t*-test was conducted to assess whether the antenatal stress manipulation altered gestation outcomes as expressed in the number of pups delivered per litter. The test did not indicate a significant difference in litter size between dams that underwent the stress procedure during gestation and naïve dams (*t*_(28)_ = 0.51, *p* = 0.61, *ns*; see **Fig S1A**).

*Dams' locomotor activity*

Dams' locomotor activity was assessed following parturition, on postnatal day (PND) 1, using the locomotion index in the open field test. Dams were individually placed at the center of an empty square arena (40 × 40 × 40 cm), surrounded by Perspex opaque walls. Their behavior was video-recorded for 5 min and later automatically coded using the Viewer software (Biobserve GmbH, Bonn, Germany). The arena was thoroughly cleaned between sessions. Locomotor activity was expressed as the percentage of time that the dam was moving in a velocity above 0.1 pixel/sec; this index represents general activity and serves as a control measure to ascertain that dams' motor functioning was intact^1^.

An independent samples *t*-test did not indicate a significant difference in locomotor activity between dams that underwent the stress procedure during gestation and naïve dams (*t*_(28)_ = 0.35, *p* = 0.73, *ns*; see **Fig S1B**).

*Dams' weight*

A mixed-design ANOVA was conducted to assess whether antenatal stress (naïve / stress) and pharmacological treatments (saline / escitalopram / shan-zha) altered the fluctuations in dams' weight between PND 1 and PND 20 (overall 7 assessments; approximately one per three days).

The analysis indicated that sphericity could not be assumed (Mauchly's *W* = 0.087, χ^2^_(20)_ = 53.1, *p* < 0.001), and, therefore, the Greenhouse-Geisser's degrees of freedom correction was employed.

The analysis revealed a significant effect for the time assessment (*F*_(3.5, 83.3)_ = 47.6, *p* < 0.0001; see **Fig S1C**). *Post hoc* analysis using the Bonferroni correction indicated that dams' weight on PND 1 was significantly lower, when compared to all other PNDs (*p* < 0.001 in all contrasts except for PND 20 where *p* = 0.005). Dams' weight on PND 4 was significantly lower when compared to PNDs 7, 10 and 14 (*p* < 0.001 in all contrasts); and dams' weight on PND 20 was significantly lower when compared to PNDs 7, 10, 14 and 17 (*p* < 0.001 in all contrasts). Taken together, this main effect suggests that in all groups weight fluctuated in an inverted-U curve with lower weights following parturition and upon weaning.

Importantly though, the analysis did not reveal a time × stress interaction effect (*F*_(3.5, 83.3)_ = 2.37, *p* = 0.067), suggesting only a non-significant trend for lower weights in stressed dams on PND 1 that entirely ebbed later on. Overall, this suggests that the stress manipulation did not significantly alter dams' weight.


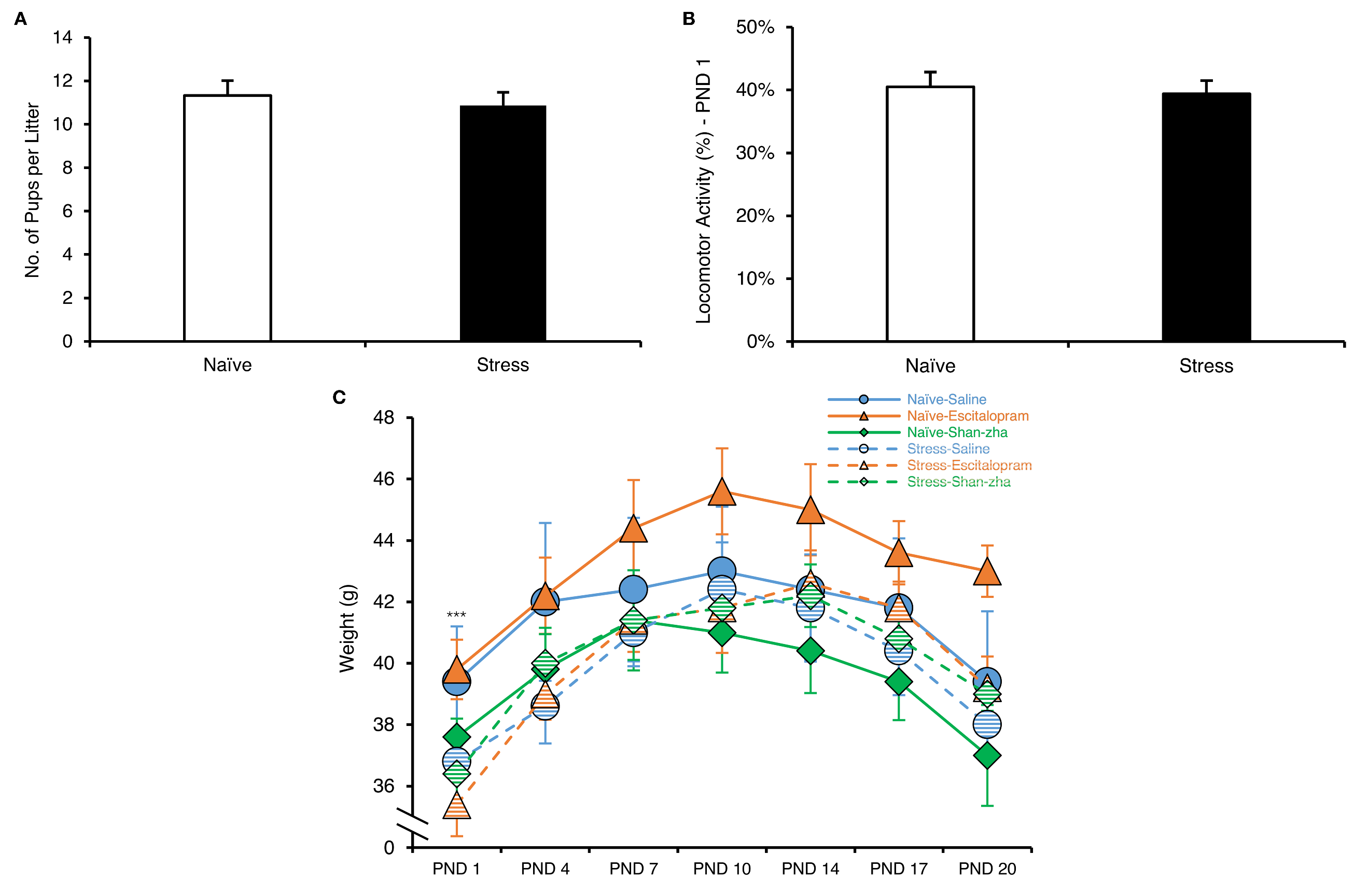

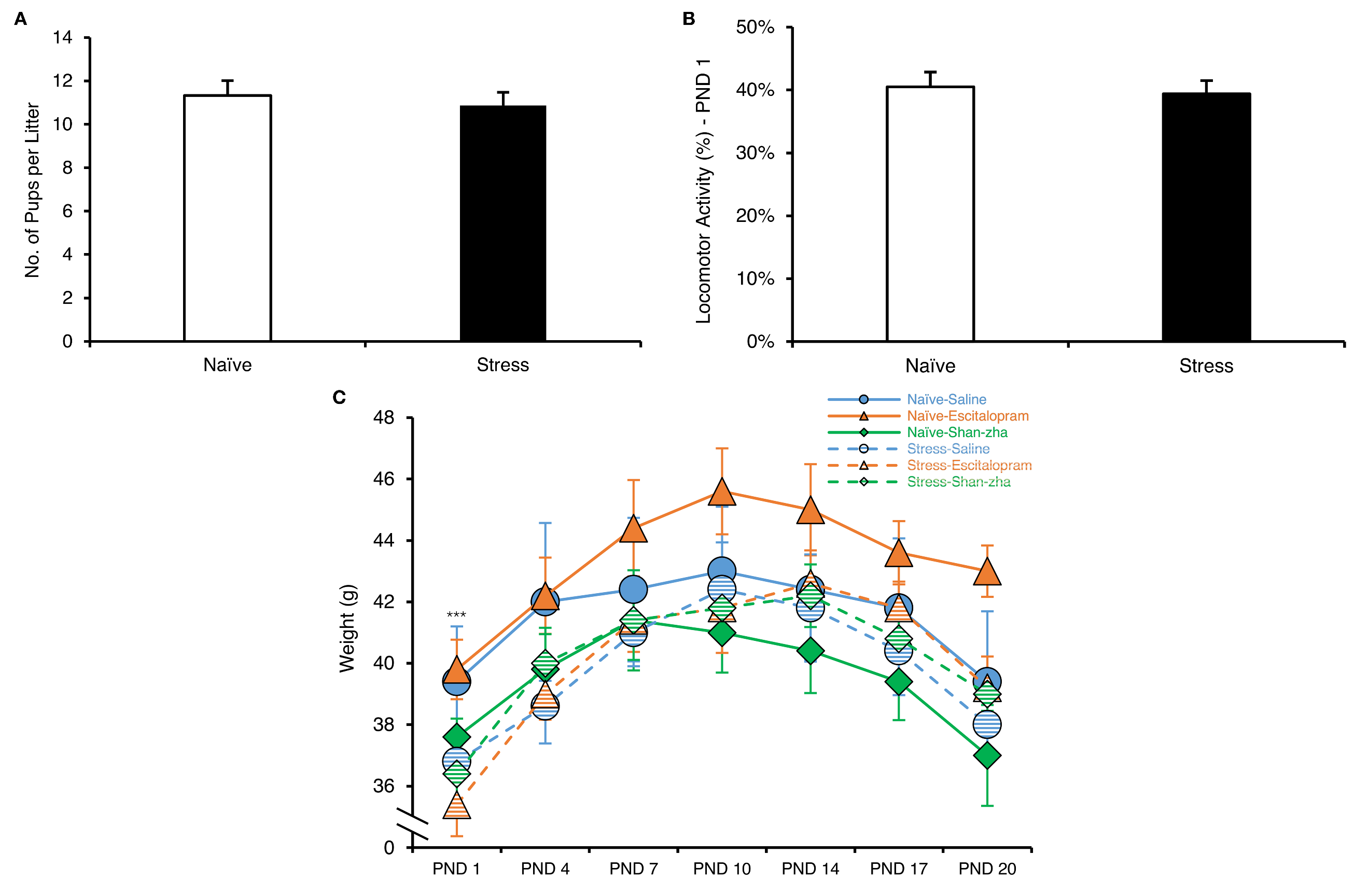

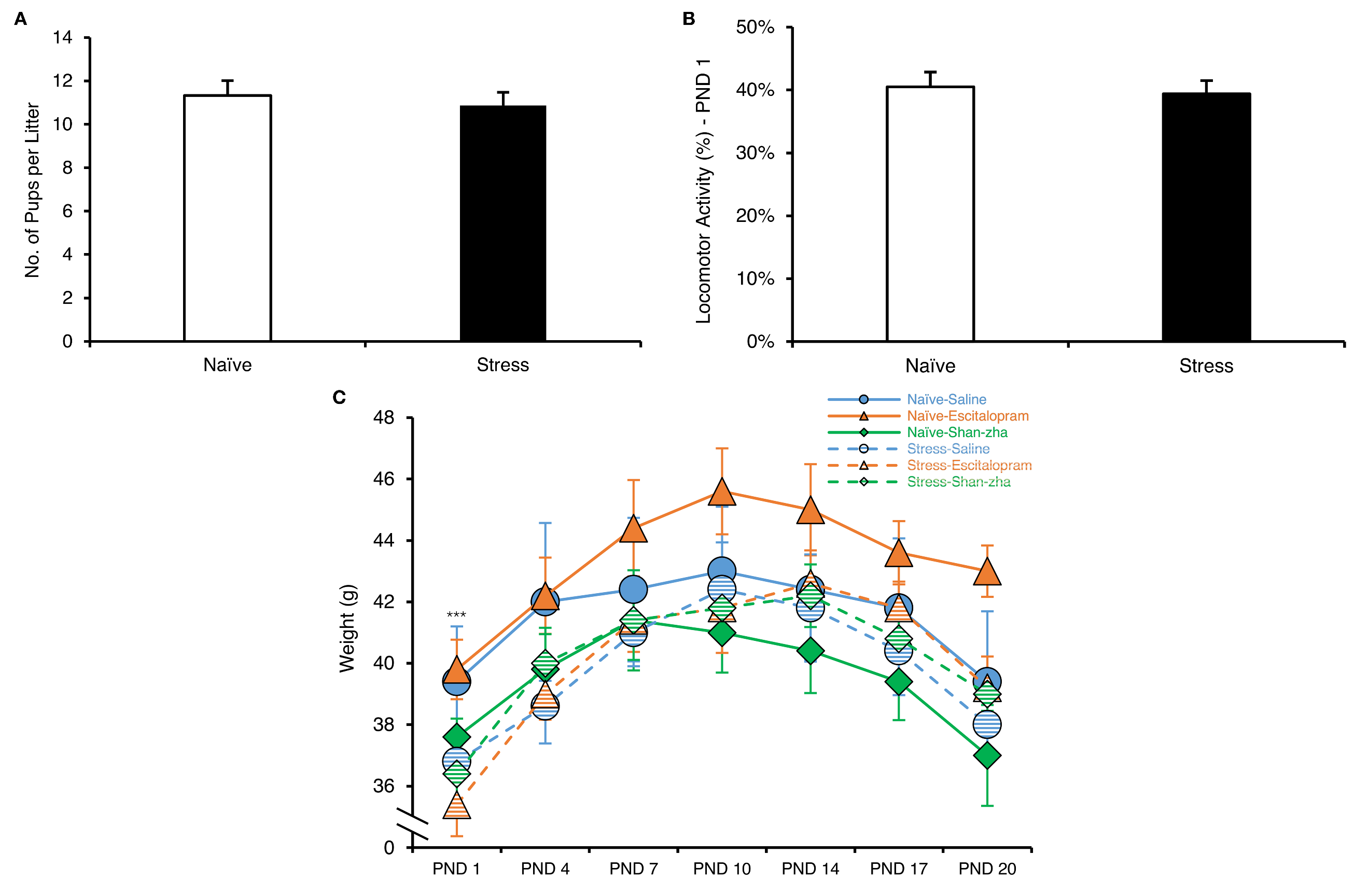
**Fig S1. Assessments of possible effects of antenatal stress on litter size, locomotion and weight in dams.** No differences were found between the naïve-stress groups in litter size (A) and locomotor activity (B). Vis-à-vis dams' weight, a significant effect for time was found, suggesting lower weights on PND 1 and 20, without further significant effects for stress or treatment. Results are expressed as means ± SEM. *N* = 30. *** *p* < 0.005 when compared to all other PNDs.

**Appendix B**

**Assessments of the effects of pups' sex**

*Pups anxiety-like behavior on PND 21*

A three-way ANOVA (2 × 3 × 2) on pups' anxiety-like behavior (*i.e.*, the percentage of time spent on the open arms of the maze) on PND 21 was conducted, with stress (naïve / antenatal stress), treatment (control / escitalopram / shan-zha) and sex (male / female) as the independent variables. No significant effects involved differences between male and female mice, including no main effect for sex (*F*_(1,209)_ = 0.53, *p* = 0.47), and no stress × sex (*F*_(1,209)_ = 1.55, *p* = 0.21), treatment × sex (*F*_(2,209)_ = 0.68, *p* = 0.51), and stress × treatment × sex (*F*_(2,209)_ = 1.41, *p* = 0.25) interaction effects (see **Table S1** for descriptive statistics).

| **Table S1**  Descriptive statistics on pups' anxiety-like behavior on PND 21 | | | |
| --- | --- | --- | --- |
|  |  | Sex | |
| Stress | Treatment | Male | Female |
|  | Control | 0.339 (0.148) | 0.266 (0.107) |
| Naïve | Escitalopram | 0.295 (0.168) | 0.270 (0.112) |
|  | Shan-zha | 0.334 (0.150) | 0.316 (0.134) |
|  | Control | 0.144 (0.087) | 0.177 (0.122) |
| Antenatal Stress | Escitalopram | 0.274 (0.158) | 0.331 (0.198) |
|  | Shan-zha | 0.344 (0.140) | 0.284 (0.160) |
| ***Note.*** The results are expressed as means and standard deviations for the percentage of time spent on the open arms of the maze. *n* = 14–22 mice per cell. *N* = 221. | | | |

*Pups free SERT*

A three-way ANOVA (2 × 3 × 2) on pups' free SERT concentrations was conducted, with stress (naïve / antenatal stress), treatment (control / escitalopram / shan-zha) and sex (male / female) as the independent variables. No significant effects involved differences between male and female mice, including no main effect for sex (*F*_(1,78)_ = 0.05, *p* = 0.83), and no stress × sex (*F*_(1,78)_ = 0.33, *p* = 0.56), treatment × sex (*F*_(2,78)_ = 0.75, *p* = 0.47), and stress × treatment × sex (*F*_(2,78)_ = 1.92, *p* = 0.15) interaction effects (see **Table S2** for descriptive statistics).

| **Table S2**  Descriptive statistics on pups' free SERT | | | |
| --- | --- | --- | --- |
|  |  | Sex | |
| Stress | Treatment | Male | Female |
|  | Control | 0.331 (0.115) | 0.318 (0.115) |
| Naïve | Escitalopram | 0.304 (0.063) | 0.243 (0.066) |
|  | Shan-zha | 0.263 (0.082) | 0.402 (0.193) |
|  | Control | 0.315 (0.072) | 0.340 (0.186) |
| Antenatal Stress | Escitalopram | 0.239 (0.038) | 0.228 (0.078) |
|  | Shan-zha | 0.419 (0.190) | 0.375 (0.183) |
| ***Note.*** The results are expressed as means and standard deviations for the level of SERT free for binding in pups' assays. *n* = 6–8 mice per cell. *N* = 90. | | | |

*Considering sex as a covariate in the moderated mediation analyses*

In additional screenings, sex was entered into the conditional process analyses as a covariate. In all of the models presented in the current study we ascertained that pups' sex had neither significant effect nor contribution to the models.

**Appendix C**

**Extended delineation of the conditional process analyses**

First, we present the comprehensive statistics for the conditional process analysis presented in the main text, weighing the effect of antenatal stress on pups' anxiety-like behavior, with dams' anxiety-like behavior on PND 1 as the mediator (**Table S3**).

| **Table S3**  A conditional process analysis testing whether escitalopram and shan-zha ameliorated the transgenerational transference of the antenatal stress-induced anxiety-like behavior by altering dams' anxiety from PND 1 | | | | | | | | |
| --- | --- | --- | --- | --- | --- | --- | --- | --- |
|  | Dams' Anxiety PND 1 (*M*) | | | | Pups' Anxiety PND 21 (*Y*) | | | |
| Antecedent |  | *B* (*SE*) | | 95% CI |  | *B* (*SE*) | | 95% CI |
| Constant | *i_M_* → | 0.295*** (0.010) | | 0.276, 0.314 | *i_Y_* → | 0.075 (0.049) | | -0.022, 0.172 |
| Antenatal Stress (*X*) | *a*_1_ → | -0.175*** (0.01) | | -0.202, -0.149 | *c'* → | 0.013 (0.027) | | -0.040, 0.065 |
| Dams' Anxiety (*M*) |  |  | |  | *b*_1_ → | 0.729*** (0.181) | | 0.373, 1.085 |
| Shan-zha (*W*_1_) |  |  | |  | *b*_2_ → | 0.205*** (0.047) | | 0.113, 0.297 |
| Escitalopram (*W*_2_) |  |  | |  | *b*_3_ → | 0.195*** (0.047) | | 0.103, 0.287 |
| Stress × Shan (*X* × *W*_1_) |  |  | |  | *b*_4_ → | -0.534** (0.203) | | -0.933, -0.135 |
| Stress × Esc (*X* × *W*_2_) |  |  | |  | *b*_5_ → | -0.659*** (0.19) | | -1.035, -0.282 |
|  | *R^2^* = 43.4%  *F*_(1, 219)_ = 167.67, *p* < 0.0001 | | | | *R^2^* = 15.2%  *F*_(6, 214)_ = 6.41, *p* < 0.0001 | | | |
| *Conditional indirect effects as a function of treatment* | | | | | | | | |
| Treatment | Effect | | Boot *SE* | | 95% Boot LLCI | | 95% Boot ULCI | |
| Control | -0.128 | | 0.033 | | -0.197 | | -0.068 | |
| Shan-zha | -0.034 | | 0.025 | | -0.083 | | 0.015 | |
| Escitalopram | -0.012 | | 0.027 | | -0.062 | | 0.043 | |
| *Indices of moderated mediation* | | | | | | | | |
|  | Index | | Boot *SE* | | 95% Boot LLCI | | 95% Boot ULCI | |
| Shan-zha | 0.094 | | 0.037 | | 0.024 | | 0.167 | |
| Escitalopram | 0.115 | | 0.034 | | 0.051 | | 0.186 | |
| ***Note.*** A conditional process analysis to test whether the mediation of the effect antenatal stress (*X*) on pups' anxiety-like behavior on PND 21 (*Y*) through dams' anxiety-like behavior on PND 1 (*M*) is moderated by dams' subsequent treatment with either shan-zha (*W*_1_) or escitalopram (*W*_2_). Antenatal stress led to a significant decrease in the time dams spent in the open arms of the maze on PND 1 (*a*_1_ = -0.15, *p* < 0.0001), accounting for 43.4% of the variance; chronic treatment with either escitalopram (*b*_5_ = -0.66, *p* = 0.0007) or shan-zha (*b*_4_ = -0.53, *p* = 0.009) to lactating dams that were exposed to antenatal stress significantly moderated the association between dams' and pups' anxiety. Higher values on the anxiety variables indicate diminished anxiety-like behavior (*i.e.*, diminished percentage of time spent on the open arms of the maze). *N* = 221. *** *p* < 0.001 ** *p* < 0.01 | | | | | | | | |

Subsequently, we conducted an additional conditional process analysis to assess whether the moderated mediation is also observed when dams' anxiety-like behavior on PND 21 (rather than PND 1) is considered as the mediator.

In line with the first model, the analysis indicated that the effect of antenatal stress on pups' anxiety-like behavior was mediated by dams' anxiety-like behavior on PND 21; and, both escitalopram and shan-zha moderated the stress-induced transference of anxiety from dams to pups by soothing dams' anxiety on PND 21 (escitalopram: *B* = 0.32, *p* < 0.0001; shan-zha: *B* = 0.28, *p* < 0.0001). The indices of moderated mediation suggested that dams' treatment with either escitalopram (95% Boot CI, 0.082 to 0.198) or shan-zha (95% Boot CI, 0.071 to 0.173) ameliorated the transference of anxiety to pups in the stress group (see **Table S4** and **Fig S2**).

| **Table S4**  A conditional process analysis testing whether escitalopram and shan-zha ameliorated the transgenerational transference of the antenatal stress-induced anxiety-like behavior by altering dams' anxiety on PND 21 | | | | | | | | |
| --- | --- | --- | --- | --- | --- | --- | --- | --- |
|  | Dams' Anxiety PND 21 (*M*) | | | | Pups' Anxiety PND 21 (*Y*) | | | |
| Antecedent |  | *B* (*SE*) | | 95% CI |  | *B* (*SE*) | | 95% CI |
| Constant | *i_M_* → | 0.261*** (0.015) | | 0.276, 0.314 | *i_Y_* → | 0.213*** (0.022) | | 0.169, 0.257 |
| Antenatal Stress (*X*) | *a*_1_ → | -0.177*** (0.02) | | -0.202, -0.149 | *c'* → | -0.058** (0.019) | | -0.096, -0.019 |
| Dams' Anxiety (*M*) |  |  | |  | *b*_1_ → | 0.424*** (0.079) | | 0.269, 0.580 |
| Shan-zha (*W*_1_) | *a*_2_ → | -0.05* (0.021) | | -0.092, -0.009 |  |  | |  |
| Escitalopram (*W*_2_) | *a*_3_ → | -0.073** (0.022) | | -0.116, -0.031 |  |  | |  |
| Stress × Shan (*X* × *W*_1_) | *a*_4_ → | 0.281*** (0.029) | | 0.223, 0.339 |  |  | |  |
| Stress × Esc (*X* × *W*_2_) | *a*_5_ → | 0.323*** (0.03) | | 0.264, 0.381 |  |  | |  |
|  | *R^2^* = 48.3%  *F*_(5, 215)_ = 40.1, *p* < 0.0001 | | | | *R^2^* = 13.8%  *F*_(6, 218)_ = 17.4, *p* < 0.0001 | | | |
| *Conditional indirect effects as a function of treatment* | | | | | | | | |
| Treatment | Effect | | Boot *SE* | | 95% Boot LLCI | | 95% Boot ULCI | |
| Control | -0.075 | | 0.017 | | -0.110 | | -0.044 | |
| Shan-zha | 0.044 | | 0.012 | | 0.023 | | 0.070 | |
| Escitalopram | 0.062 | | 0.016 | | 0.033 | | 0.096 | |
| *Indices of moderated mediation* | | | | | | | | |
|  | Index | | Boot *SE* | | 95% Boot LLCI | | 95% Boot ULCI | |
| Shan-zha | 0.119 | | 0.026 | | 0.071 | | 0.173 | |
| Escitalopram | 0.137 | | 0.029 | | 0.082 | | 0.198 | |
| ***Note.*** A conditional process analysis to test whether the mediation of the effect antenatal stress (*X*) on pups' anxiety-like behavior on PND 21 (*Y*) through dams' anxiety-like behavior on PND 21 (*M*) is moderated by dams' previous treatment with either shan-zha (*W*_1_) or escitalopram (*W*_2_). Antenatal stress led to a significant decrease in the time dams spent in the open arms of the maze on PND 21 (*a*_1_ = -0.18, *p* < 0.0001); however, chronic treatment with either escitalopram (*a*_5_ = 0.32, *p* < 0.0001) or shan-zha (*a*_4_ = 0.28, *p* < 0.0001) to lactating dams significantly moderated this association; dams' anxiety-like behavior on PND 21 was significantly associated in the model with pups' anxiety-like behavior on PND 21(*b*_1_ = 0.43, *p* < 0.0001), accounting for 13.8% of the variance. Higher values on the anxiety variables indicate diminished anxiety-like behavior (*i.e.*, diminished percentage of time spent on the open arms of the maze). *N* = 221. *** *p* < 0.001 ** *p* < 0.01 * *p* < 0.05 | | | | | | | | |

**
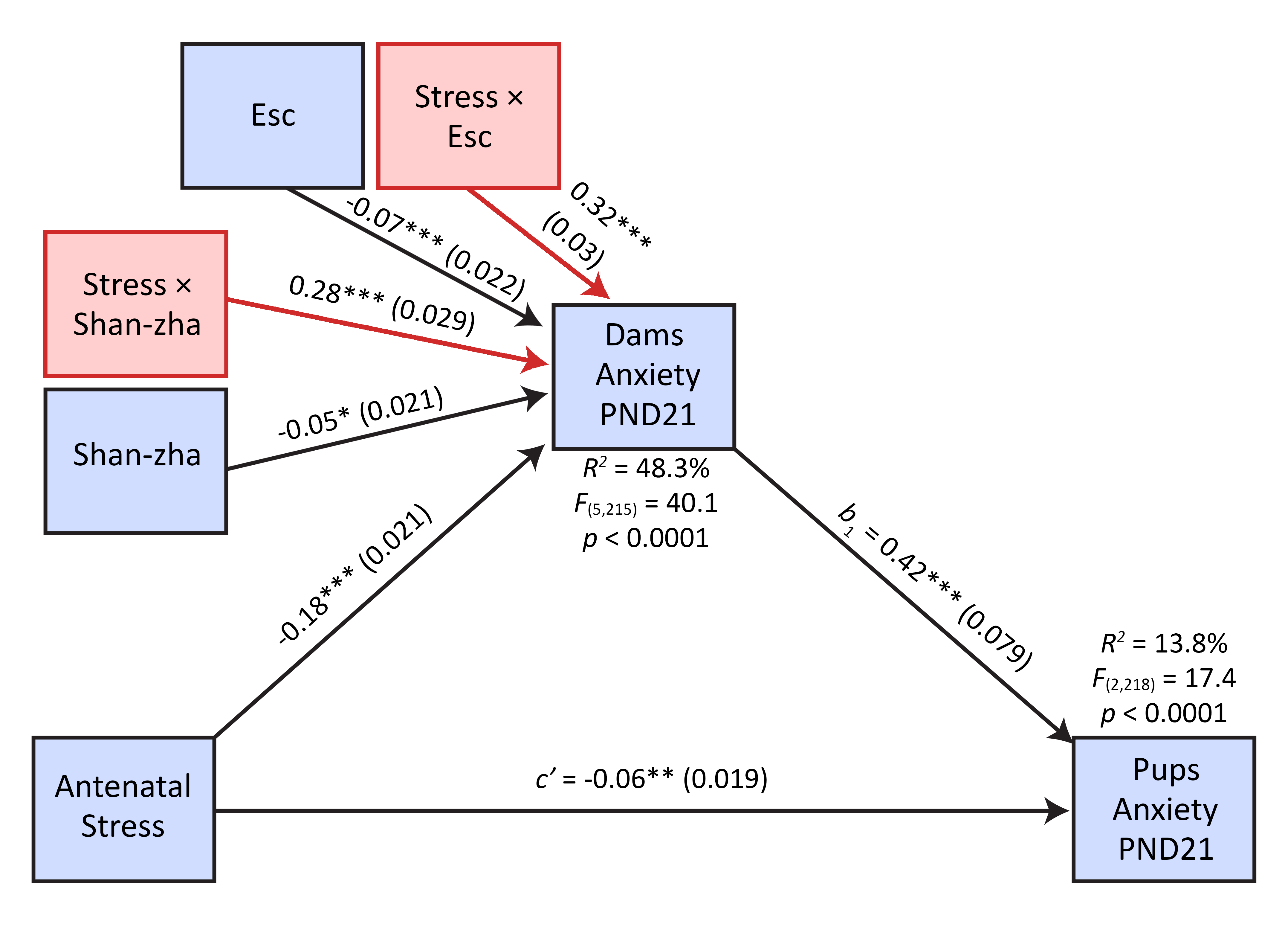
**

**Fig S2. A graphical depiction of the moderated mediation analysis with dams' anxiety-like behavior on PND 21 as the mediator.** *N* = 221. *** *p* < 0.001 ** *p* < 0.01 * *p* < 0.05

| **Table S5**  A conditional process analysis testing whether antenatal stress exacerbated pups' exposure to escitalopram via lactation | | | | | | | | |
| --- | --- | --- | --- | --- | --- | --- | --- | --- |
|  | Dams' free SERT (*M*) | | | | Pups' free SERT (*Y*) | | | |
| Antecedent |  | *B* (*SE*) | | 95% CI |  | *B* (*SE*) | | 95% CI |
| Constant | *i_M_* → | 1.066*** (0.521) | | 0.962, 1.170 | *i_Y_* → | 0.868*** (0.12) | | 0.621, 1.114 |
| Escitalopram (*X*) | *a*_1_ → | -1.061*** (0.09) | | -1.246, -0.875 | *c'* → | 0.037 (0.134) | | -0.229, 0.304 |
| Dams' free SERT (*M*) |  |  | |  | *b*_1_ → | 0.110 (0.107) | | -0.104, 0.323 |
| Antenatal stress (*W*) |  |  | |  | *b*_2_ → | -0.249* (0.121) | | -0.489, -0.009 |
| Treat × Stress (*X* × *W*) |  |  | |  | *b*_3_ → | 0.393** (0.125) | | 0.145, 0.642 |
|  | *R^2^* = 59.4%  *F*_(1, 88)_ = 128.7, *p* < 0.0001 | | | | *R^2^* = 25%  *F*_(4, 85)_ = 7.09, *p* < 0.0001 | | | |
| *Conditional indirect effects as a function of antenatal stress exposure* | | | | | | | | |
| Treatment | Effect | | Boot *SE* | | 95% Boot LLCI | | 95% Boot ULCI | |
| Naïve | -0.116 | | 0.122 | | -0.372 | | 0.105 | |
| Antenatal stress | -0.534 | | 0.129 | | -0.782 | | -0.283 | |
| *Index of moderated mediation* | | | | | | | | |
|  | Index | | Boot *SE* | | 95% Boot LLCI | | 95% Boot ULCI | |
| Antenatal stress | -0.417 | | 0.121 | | -0.644 | | -0.172 | |
| ***Note.*** A conditional process analysis to test whether the mediation of the effect dams' treatment with escitalopram (*X*) on pups' escitalopram exposure (*Y*; manifested as free SERT concentrations in the biochemical assessment) through dams' escitalopram concentrations in the serum (*M*) is moderated by dams' exposure to stress during the antenatal period (*W*). *N* = 90. *** *p* < 0.001 ** *p* < 0.01 * *p* < 0.05 | | | | | | | | |
